# Chicago Medical Society

**Published:** 1869-09-15

**Authors:** 


### SOCIETY REPORTS.

### *Chicago Medical Society.*

FRIDAY EVENING, July 23, 1869.

Regular meeting Chicago Medical Society, President Bogue in the chair. Minutes of the last meeting were read and approved.

Proposals for new members being in order, Dr. F. W. Coffin was recommended by Drs. C. T. Parkes and Quales, and was referred to board of censors.

Reports of special and standing committees being in order, Dr. Quales read a lengthy, but interesting, report of the sanitary condition of the North Division of the city. Remarks were made by Drs. Reed and others, when the report was accepted and ordered placed on file.

Reports of cases and exhibition of pathological specimens being next in order, Dr. Reed remarked that he had been called to a number of cases of cholera infantum. A few were accompanied by convulsions. While he had avoided mercurials in some cases, he had found them of positive advantage in others; that he usually used them in the form of sub. muriate, combined with very minute quantities of morphia. He spoke favorably of bismuth, sub. nitras and the chalk mixture. After further remarks upon children, he stated that he had been called to a severe case of cholerine in an adult, which presented all the symptoms of Asiatic cholera, and would have been diagnosed as such in cholera season. The patient recovered.

Dr. Marguerat asked, in the treatment of cholera morbus, the propriety of using stimulants; that he had seen much good derived from the use of burned brandy, and this year, among children, had received special benefit from pepsin; thought morphia should be given to infants with exceeding caution.

Dr. Fitch remarked that for years he had given morphia to children, often combined with calomel and bismuth, always placed dry upon the tôngue, and had seen no fatal consequences of narcotism, as described by the other gentlemen. Where the gums were tumified, he was in favor of scarifying them early. Gave as little liquid as possible. Used sinapisms to the epigastrium.

Dr. Avery stated that he used, in cholera infantum, a powder containing hydrarg. cum. cretæ and pul. doveri camphorata, the dose being adapted to the age of the child, with excellent effect.

Dr. Wanzer remarked that he had seen rather an unusual number of cases of cholera infantum this year; that most of them had recovered under very mild treatment. Where the discharges were painless, frequent and watery, one grain of calomel, divided into eight powders, one of which, given every two or three hours, was usually all-sufficient to cure the child, when seen in the onset. Where the discharges had continued several days, with vomiting, half a grain of bismuth, sub. nitras, was added to each powder of the mercurial, alternating with limewater and sweet milk, as food and medicine, prohibiting the use of water and other liquids, excepting its natural nourishment. Had seen a number of cases with dysenteric symptoms, with abdominal pain and tenesmus; the best treatment found for this was, combined with the calomel, minute quantities of pul. opii and plumbi acetas. Sinapisms to the epigastrium and bowels were not forgotten. He could bear testimony with Dr. Marguerat to the therapeutical value of pepsin in those chronic cases. After the discharges became modified by the calomel, there was often a degree of emaciation, a want of digestion and healthy assimilation, which function the pepsin seems to aid in re-establishing, alternating with this small portions of the best undiluted port wine. Had seen a number of cases of active cholera morbus; one case, a lady æt. 50, who had all the premonitory symptoms of Asiatic cholera. Calomel, morphia and bismuth were given internally, warmth and revulsives were applied to the surface. Stated in our last visitation of cholera he found it difficult to obtain medicines to arrest the persistant vomiting; finally administered a decoction of composition powder. Saved one patient with it in collapse. A drachm of the powder to one teacupful of boiling water, sweetened with loaf sugar, a teaspoonful to be given every quarter of an hour after vomiting until relieved.

On motion, Society adjourned. HIRAM WANZER, *Secretary.*

---

CHICAGO, FRIDAY EVENING, Aug. 6, 1869.

Regular meeting Chicago Medical Society, President Bogue in the chair.

Minutes of the last meeting were read and approved.

Proposals and election of new members being in order, the board of censors reported the case of Dr. Coffin favorable, whereupon he was balloted for and elected.

The Society now proceeded to the discussion appointed for the evening, viz: The pathology and best treatment for chlorosis.

Dr. Merriman stated: Chlorosis is a disease occurring usually in young girls at the age of puberty, though it has been met with in both sexes, and at all periods of life. Among the earlier symptoms are languor, lassitude, depression of spirits, nervousness, constipation, loss of appetite and disturbance of the catamenia. As the disease advances, these become more marked; the functions of digestion and assimilation become greatly disordered, with an abnormal appetite; a craving for unnatural food frequently exists; severe nervous disorders set in—headaches, neuralgias, hysteria, dyspnœa, cough, palpitation, oedema and anasarca often are present; in many cases emaciation becomes extreme, and the patient dies of phthisis pulmonalis. The appearance of a patient suffering from chlorosis is very noticeable. The countenance has a pale, waxen, greenish hue; the eyeballs seem to be enlarged and to stand out very prominently; the lips and mucus membrane are pale, and there is every appearance of anemia. So marked is this symptom, that many physicians recognize no difference between chlorosis and anemia; but there is a difference. In anemia there is a deficiency of the red corpuscles of the blood (not the white), and of the fibrin. In the blood of chlorosis there is a deficiency of both red and white corpuscles, while the fibrin exists in normal amount. Chlorosis is regarded by the best authors of the present age as a disease of the ganglionic system of nerves. The predisposing causes are age and sex. Among the exciting causes are—1st, depressing emotions, as grief, anxiety, fear, homesickness, disappointment, &c.; 2d, prolonged, fatiguing mental labor; 3d, deprivation of light, fresh air, exercise, good food and sleep; 4th, shocks to the ganglionic nervous system, as when the menses are suddenly stopped through exposure to cold or wet. In the treatment we need to surround the patient with good hygienic influences, cheerful society, amusements; to regulate the bowels by warm laxatives, as aloes; to assist digestion by the acid and bitters tonics; to sooth the nervous system by hyoscyamus, valerian, &c.; to fortify by other nerve tonics, as the shower-bath, electricity, strychnine, arsenic, and, when the digestive system will bear it, iron.

Dr. Fitch accords with the opinion of Dr. Merriman in the symptoms of the disease; believes there is a diminution in the red corpuscles of the blood and an increase in the white, a want of nerve force and tonicity. That the primary source of the disease may be the nerve centres; that nerve stimulants and tonics are indicated. Both sexes and all ages are prone to it. The treatment should be strongly supporting, to re-establish digestion and healthy assimilation, that exalt the vital actions, that give tone and energy to the impaired nerve centres and increase the red corpuscles of the blood; for this, has found a most efficient tonic in the phosphate of iron and quinia.

Dr. Parkes stated that most needed were medicines to restore the quality and condition of the blood and the primary re-establishment of all the digestive and assimilative processes.

Dr. Reid stated, in chlorosis there was anemia, a want of red corpuscles in the blood. The primary and active cause was defective nutrition, a disturbance in digestion and all the assimilative powers. The defective vitality may be ascribed to the foregoing causes. Thinks other medicines beside iron are called for in the primary treatment of the disease, viz., the oloetic purgatives with ferri sulphas; also, quinia in small doses. The shower-bath need not be neglected, followed by brisk friction. To strengthen the nerve centres and restore the red corpuscles of the blood, some of the ferruginous tonics are now wanted to complete the cure.

Dr. Paoli stated that the pathology of chlorosis was frequently obscured by a variety of causes; believes the often disturbed uterine function is a fruitful cause of the disease; that organic disease of the heart and tubercular deposit in the lungs might develop the disease. The treatment should be strongly supporting, the patient surrounded with the choicest hygenic influences.

On motion, Society adjourned. Hiram Wanzer, *Secretary.*

---

Friday Evening, August 20, 1869.

Regular meeting of the Chicago Medical Society. In the absence of President Bogue, Dr. Loverin was appointed to act, for the evening, as Chairman. Reports of cases and exhibition of pathological specimens being in order, Dr. Loverin, presented a fragment of inferior maxillary bones from a child four years old. He stated it followed scarlatina; that there was a want of plasticity of the blood; there was pus formation all over the surface of the body. The abscess he had evacuated with the lancet. Had given the child, internally, potassa chlorate and tinct. ferri muriatis. Locally had applied a lotion of soda chloride. The child had recovered.

Dr. Avery reported case of lingering labor. She had irregular uterine contractions for three days. Fearing the exhaustion of the patient a hot sitz bath was given, together with artificial dilation of the os uteri. In a few hours complete dilation was accomplished, and she was safely delivered of a male child.

Dr. Paoli disapproved of artificial irritation, where the os was tardy in dilating. He was accustomed to give one-fourth grain Ant. Pot. tartratis, with ten grains potassa nitras every half-hour, using also hot sitz bath. He condemns all digital manipulation until complete dilatation of the os.

Dr. Loverin agreed with his predecessor; believes, by artificial irritation puerperal peritonitis may supervene; that all those cases should come under the head of vis medicatrix natura.

Dr. Wanzer stated the case related by Dr. Avery was one of interest to him. He had followed the procedure of the Dr. for several years, where the case was tedious and the os tardy in dilating, by occasionally passing the index finger around its circumference and gently dilating it; he had never seen a case of metro peritonitis or other accident follow such manipulations. From the promotion of rest and relaxation of the os, one-fourth grain Ant. Pot. tartratis combined with ten grains doveri pulveris, will usually secure some refreshing sleep, together with relaxation of the womb and soft parts, and upon awaking the pains will return with renewed vigor. He had also seen labor facilitated by forcible pressure upon the perineum, in the absence of pain. He stated, on the 13th of June, ult., he was called to a young lady in labor with her first child. She had suffered active labor pains for twelve hours and was, up to my arrival, under the care of an inexperienced midwife. Upon digital examination the breech of the child was found occupying a part of the excavation. At the center of the part presenting was a deep cavity, surrounded by lacerated and broken-down tissue, which was diagnosed as the anus of the child. Our opinions were this petticoat doctor had whiled away those long hours making tractions at that point, which was true. Passing the hand between the parts of the mother and child, with the index finger the groin was easily reached. At this juncture the pains subsided. Imitating them by gentle tractions, in about three-quarters of an hour she was delivered of a living child. Upon inspection, all the structures surrounding the anus were a blackened, pulpified mass, easily brokon down by the finger. Both external and internal sphincter-ani muscles were destroyed. Both mother and child have made an excellent recovery, and I am creditably informed the child has perfect control over the sphincters.

Dr. W. also would ask the Society the best method of vaccination; that in the month of May last he vaccinated 230 children, and in June but 17. Every precaution was used in selecting the purest matter the city affords, but in at least six per cent. of all vaccinated, alarming symptoms followed. Some returned with arms tumefied twice their normal size, phlegmonous or erysipelatous inflammation around the periphery of the point vaccinated, varying from two inches to the involvement of the whole arm. Others with excavated and discharging ulcers with steep edges, one or two of which did not granulate or cicatrize for two months. There was high febrile action manifested in all those cases; most of them, seen early, yielded kindly to mercurial alteratives, and diaphoretics, and local anodyne and saturnine lotions. Most of the children were of foreign birth. In searching the cause of the phenomenon all those children were found of an aplastic diathesis; some living in streets and alleys with deficient drainage, in illy-ventilated apartments, imperfectly fed and clothed, sleeping upon the floor, breathing, not only their own exhalations, but the pestiferous atmosphere of the neighborhood. With all those unhappy circumstances, all escaped without loss of limb or life.

